# Frequency-Unspecific Effects of θ-tACS Related to a Visuospatial Working Memory Task

**DOI:** 10.3389/fnhum.2017.00367

**Published:** 2017-07-12

**Authors:** Maria-Lisa Kleinert, Caroline Szymanski, Viktor Müller

**Affiliations:** ^1^Center for Lifespan Psychology, Max Planck Institute for Human Development Berlin, Germany; ^2^Department of Education and Psychology, Freie Universität Berlin Berlin, Germany; ^3^School of Mind and Brain, Humboldt-Universität zu Berlin Berlin, Germany

**Keywords:** working memory, central executive, cortical oscillations, theta phase synchronization, tACS

## Abstract

Working memory (WM) is crucial for intelligent cognitive functioning, and synchronization phenomena in the fronto-parietal network have been suggested as an underlying neural mechanism. In an attempt to provide causal evidence for this assumption, we applied transcranial alternating current stimulation (tACS) at theta frequency over fronto-parietal sites during a visuospatial match-to-sample (MtS) task. Depending on the stimulation protocol, i.e., in-phase, anti-phase or sham, we anticipated a differential impact of tACS on behavioral WM performance as well as on the EEG (electroencephalography) during resting state before and after stimulation. We hypothesized that in-phase tACS of the fronto-parietal theta network (stimulation frequency: 5 Hz; intensity: 1 mA peak-to-peak) would result in performance enhancement, whereas anti-phase tACS would cause performance impairment. Eighteen participants (nine female) received in-phase, anti-phase, and sham stimulation in balanced order. While being stimulated, subjects performed the MtS task, which varied in executive demand (two levels: low and high). EEG analysis of power peaks within the delta (0.5–4 Hz), theta (4–8 Hz), alpha (8–12 Hz), and beta (12–30 Hz) frequency bands was carried out. No significant differences were observed between in-phase and anti-phase stimulation regarding both behavioral and EEG measurements. Yet, with regard to the alpha frequency band, we observed a statistically significant drop of peak power from pre to post in the sham condition, whereas alpha power remained on a similar level in the actively stimulated conditions. Our results indicate a frequency-unspecific modulation of neuronal oscillations by tACS. However, the closer participants’ individual theta peak frequencies were to the stimulation frequency of 5 Hz after anti-phase tACS, the faster they responded in the MtS task. This effect did not reach statistical significance during in-phase tACS and was not present during sham. A lack of statistically significant behavioral results in the MtS task and frequency-unspecific effects on the electrophysiological level question the effectiveness of tACS in modulating cortical oscillations in a frequency-specific manner.

## Introduction

In line with Baddeley’s multicomponent model (Baddeley and Hitch, 1974; Baddeley, 2000), working memory (WM) refers to the temporary storage as well as manipulation of information for goal-directed behavior. Neurobiological and neuroimaging findings over the last decades have conveyed the idea that WM might depend on specific anatomical structures, including prefrontal and parietal regions (Goldman-Rakic, 1988; Curtis and D’Esposito, 2003; Bledowski et al., 2009). However, the premise of anatomical localization as a solid theoretical basis for a system as pervasive as WM has been criticized (Baddeley, 2012). Consequently, in recent years progressively more studies have shifted their interest from an exclusive “where” approach toward the “when” of WM processes and/or components (Sauseng et al., 2005, 2010; Jensen et al., 2007; Klimesch et al., 2010; Roberts et al., 2013; Roux and Uhlhaas, 2014). But which mechanisms characterize the temporal dynamics of WM? How does the brain simultaneously orchestrate activity between distant neural networks?

An answer to these questions may come from research conducted on cortical oscillations, a phenomenon ubiquitous in the human brain. Brain oscillations in different frequency bands have proven crucial for attentional as well as perceptual processes (Vanrullen and Dubois, 2011). Oscillations within the theta frequency band in particular have been associated with a wide range of behavioral processes, such as orienting reflex, attention, arousal, and memory, conditioning and learning, including binding and information processing mechanisms (Buzsáki, 2005). Similarly, enhanced oscillatory activity at delta frequency during cognitive tasks may be an indicator of attention and task demand (Harmony et al., 1996; McEvoy et al., 2001; Müller et al., 2009), as well as of response production and inhibition (Müller and Anokhin, 2012; Lavallee et al., 2014). In contrast to delta and theta frequency, alpha and beta rhythms show tendencies to reduce or to desynchronize during perceptual and memory tasks (Pfurtscheller and Lopes da Silva, 1999).

The synchronization of frequency-specific oscillatory activity between remote cortical networks may be understood as a ‘fingerprint’ of neural computations necessary for cognitive processes (Siegel et al., 2012). Oscillations ranging from lower (0.05 Hz) to higher frequencies (500 Hz) have been associated with specific cognitive/behavioral states (Wang, 2010) and synchrony within, as well as between, frequency bands has been reported to underlie process binding and large scale integration in general (Varela et al., 2001; Uhlhaas et al., 2008; Klimesch et al., 2010). Interestingly, there seems to be a relationship between the extension of functional integration and the synchronization frequency, i.e., lower frequencies, such as theta (4–8 Hz) and alpha (8–13 Hz) enable long-range fronto-parietal interactions, whereas higher frequencies (e.g., gamma, 30–200 Hz) seem to be particularly suitable for local, short-range integration (von Stein and Sarnthein, 2000).

With regard to WM maintenance, a recent review of EEG, MEG, and ECoG studies proposed distinct functional roles for neural oscillations at theta, alpha and gamma frequency. Gamma-band activity might be involved in maintaining WM information, whereas theta oscillations seem to play a key role in the temporal organization of sequentially ordered WM items (Roux and Uhlhaas, 2014). According to the inhibition-timing hypothesis (Klimesch et al., 2007), event-related synchronization in the alpha frequency band reflects top–down inhibitory control and timing processes of task-irrelevant cortical regions. On the other hand, event-related desynchronization indicates a gradual release of inhibition (Klimesch et al., 2007). Nonetheless, alpha oscillations have not only been associated with inhibitory processes of task-irrelevant material but also executive control of behavior and active task-relevant processing (Palva and Palva, 2011). Very little is known about the role of oscillations in the actual manipulation of WM content. Within Baddeley’s multicomponent model, the modality-free central executive would be responsible for online manipulation as well as temporal coding or sequencing of WM content, updating of information, interference control, and also attentional and monitoring processes (Smith and Jonides, 1999). Thus, reluctance to investigate the central executive arises from the complications posed by its fractioned and distributed nature (Baddeley, 2012).

Nonetheless, a direct involvement of fronto-parietal theta phase coupling in central executive control mechanisms of WM has been suggested (Sauseng et al., 2005, 2010; Mizuhara and Yamaguchi, 2007). This interregional synchrony may even constitute an electrophysiological signature of the fronto-parietal control network (Dosenbach et al., 2008); an idea that is consistent with the finding that theta phase coupling is generally more spread across the brain compared to phase synchronization within the gamma range (Buzsáki, 2006). Such a spread may ensure the simultaneous activation of distinct local assemblies, each synchronized in the gamma band (Fell and Axmacher, 2011). Since most studies conducted so far on the topic of WM functioning have been correlational, the question of causality remains unsolved: is fronto-parietal theta phase synchronization a mere by-product of executive control in WM or does it have a causal function in “gating” the temporal window of integration?

One possible way of addressing this question is the use of transcranial alternating current stimulation (tACS), a relatively new and promising tool within the field of non-invasive brain stimulation which remains to some extent controversial (Kunz et al., 2016; Rjosk et al., 2016). TACS, the external application of weak sinusoidal electrical currents, is believed to entrain intrinsic cortical oscillations (Antal and Paulus, 2013) and may thus pave the way to investigate causal relationships between cortical oscillations and cognition. In contrast to direct current (DC), alternating current (AC) is not constant but switches polarity between anode and cathode with a sinusoidal waveform. *In vitro* and *in vivo* animal studies have suggested periodic modulation of transmembrane potentials (neural excitability) and entrainment of ongoing neural rhythms (shifts in spike-timing and firing) as key mechanisms of tACS (Fröhlich and McCormick, 2010; Reato et al., 2013).

However, the precise mechanisms of tACS are still debated: a recent tACS-fMRI study suggested that tACS does not necessarily cause its strongest effects underneath the stimulation electrodes, but in anatomically distant, yet functionally connected regions (Cabral-Calderin et al., 2016). Frequency-specificity has been reported in various empirical studies (Feurra et al., 2011; van Driel et al., 2015; Santarnecchi et al., 2016), even though there also is evidence for the method’s frequency-unspecific effects (e.g., Brignani et al., 2013). Comparing pre- and post-stimulation EEG recordings, the application of tACS within participants’ individual alpha peak frequency (iAPF), led to a frequency-specific amplitude enhancement of endogenous oscillations (Zaehle et al., 2010). Neuling et al. (2013) replicated this finding and showed that the alpha amplitude enhancement outlasted the duration of stimulation for at least 30 min. Recently, Kasten et al. (2016) reported aftereffects of α-tACS up to a duration of 70 min. A study conducted by Polanía and colleagues has been particularly interesting with regard to the role of theta oscillations in the maintenance of WM. The authors applied θ-tACS with varying phase-lag between left frontal and parietal regions (return electrode: Cz). Reaction times (RTs) during a delayed letter discrimination task were shorter when fronto-parietal stimulation was ‘synchronized,’ whereas participants’ performance deteriorated in the ‘desynchronized’ condition (Polanía et al., 2012).

The importance of this result for our understanding of the neural mechanisms orchestrating WM and the uncertainties about the effects of tACS on neuronal processing motivated the current study.

With this study we aimed to replicate the results on the importance of theta oscillations for WM performance reported by Polanía et al. (2012). We applied the same stimulation protocol used by Polanía and colleagues with a different WM paradigm, namely a visuospatial match-to-sample (MtS) task originally designed by Griesmayr et al. (2014). In contrast to Polanía et al. (2012) we controlled for additional factors that might drive performance changes, i.e., direction of current flow (Thut et al., 2017) as well as current intensities.

We hypothesized that similarly to the study by Polanía et al. (2012) fronto-parietal in-phase tACS at 5 Hz would enhance participants’ performance, whereas anti-phase stimulation would similarly deteriorate their performance. We expected a particularly pronounced effect at high levels of executive demand. Moreover, we assumed that these behavioral effects would be in line with electrophysiological changes of EEG peak power values within the theta frequency band. Specifically, we hypothesized that EEG theta power would be enhanced upon active tACS (independently of the type of stimulation applied, in-phase or anti-phase) compared to sham. Of note, EEG power enhancement as an effect of tACS has been previously reported in other empirical studies (Zaehle et al., 2010; Neuling et al., 2013). In order to be able to detect possible effects in other frequency bands, we did not limit our analysis to the theta frequency band only, but also considered delta (0.5–4 Hz), alpha (8–12 Hz), and beta (12–30 Hz) frequency bands. We further hypothesized that each participant’s theta peak power, as measured before stimulation during resting EEG, would shift closer to the stimulation frequency after receiving tACS compared to sham.

## Materials and Methods

### Participants

A total number of 18 healthy subjects aged 20-29 years (*M* = 25.2, *SD* = 2.96) were recruited for the present experiment. Sample exclusion criteria included: left-handedness, age below 20 or above 29 years, history of severe medical and/or psychiatric conditions, pharmacological treatment with centrally acting drugs, non-removable metal parts of the head or implanted electronic devices, acute infection/discomfort. Furthermore, to ensure experimental blinding, only subjects being naive to transcranial electrical stimulation methods were included (Ambrus et al., 2010).

The study was approved by the ethics committee of the Deutsche Gesellschaft für Psychologie and was performed in agreement with the Declaration of Helsinki. Participants’ informed written consent was acquired. The experiment was conducted in the EEG laboratories of the Max Planck Institute for Human Development in Berlin and all subjects were monetarily compensated according to local standards. Each subject was invited to three experimental sessions, which were scheduled at least 5 days apart from each other.

### Experimental Design

**FIGURE 1:**
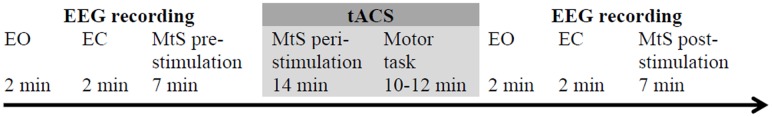
Experimental design. The time course of experimental phases is presented. EC, eyes closed; EO, eyes open; MtS, match-to-sample task.

As illustrated in **Figure 1**, the experiment consisted of three components: first, participants’ EEG was recorded before stimulation during a resting condition followed by 7 min of the delayed MtS task. Next, stimulation was turned on for 26 min. During the stimulation, participants engaged in the MtS task for 14 min and afterward completed a simple motor task for 10–12 min. Finally, stimulation was turned off and EEG was recorded again during a resting condition followed by 7 min of the MtS task. Overall, each experimental session lasted approximately 1.5–2 h. As a replication of the study by Polanía et al. (2012), sessions with different stimulation conditions were counterbalanced using a Latin square design (see Experimental Procedure).

### EEG Recording

EEG was recorded from 18 recording sites (i.e., Fp1, Fpz, Fp2, F7, F3, Fz, T7, C3, C4, T8, P7, P3, Pz, P4, P8, O1, Oz, and O2) using active Ag/AgCl electrodes mounted in an elastic cap and placed according to the international 10–20 system, with the reference electrode at the right mastoid (actiCAP, Brain Products, Munich, Germany). In order to control for eye blinks and movements, the vertical and horizontal electrooculogram (EOG) was measured. All channels were recorded with a sampling rate of 1000 Hz and a bandpass filter of 0.01-250 Hz. Electrode impedance was kept below 10 kΩ throughout the entire EEG recording.

### Experimental Procedure

**FIGURE 2:**
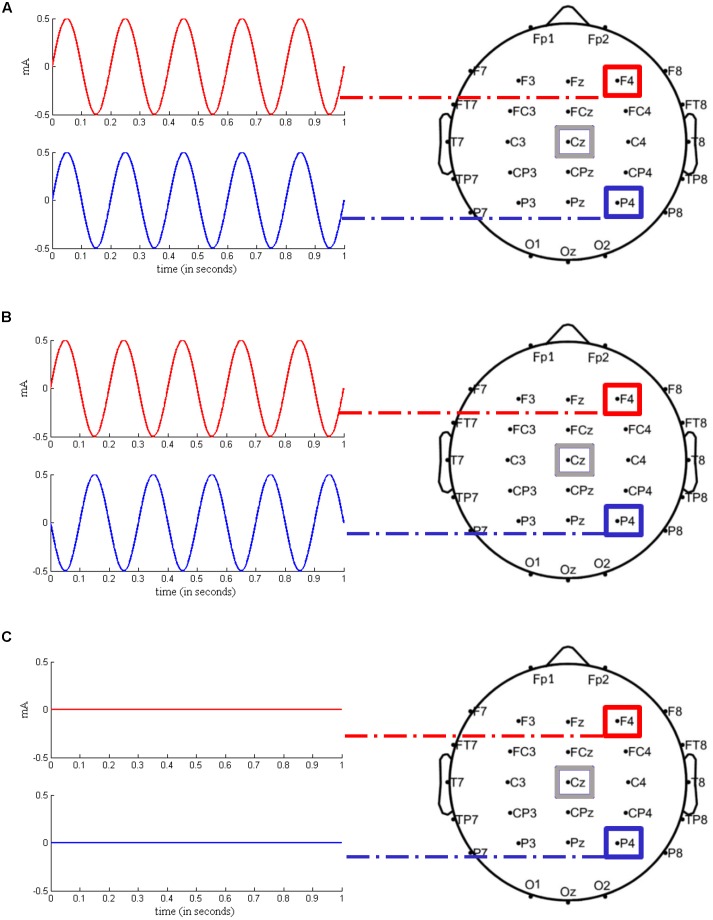
Experimental stimulation setups. **(A)** In-phase condition. Transcranial alternating current stimulation (tACS) at a frequency of 5 Hz was applied over right frontal (F4) and right parietal (P4) regions, with a 0° relative phase angle. The return electrode was located over Cz. **(B)** Anti-phase condition. tACS at a frequency of 5 Hz was applied over the same regions as in **(A)**, with a 180° relative phase angle. **(C)** Sham condition. The current was gradually ramped in during 15 s at the beginning of the stimulation phase and then gradually ramped out during 15 s until 0 mA was reached. Stimulation setups were adapted from Polanía et al. (2012) and modified respectively.

In order to rule out between-subject differences in executive control prior to stimulation, all participants completed a 1.5 h testing session 1 week before the actual experimental sessions started. During this testing session, participants completed a short version of the Raven Advanced Progressive Matrices test (Heller et al., 2006) and three different computerized short versions of complex span tasks, i.e., operation span, symmetry span, and rotation span (Foster et al., 2014), which served as measures of WM capacity. Taking into account the scores on the test battery, subjects were then pseudo-randomly assigned to one of three groups to ensure that groups did not differ in any parameter other than stimulation order. As shown in **Table 1** and identical to previous work by Polanía et al. (2012), each group consisted of six subjects (three female). In order to control for the sequence of stimulation conditions, each group received in-phase, anti-phase, and sham stimulation in a different order, resulting in a Latin square repeated measures design. In further analysis, the stimulation conditions (in-phase/anti-phase/sham) were treated as a within-subject factor. **Figure 2** illustrates the three experimental conditions: (1) in-phase condition (tACS at 5 Hz with a relative phase difference of 0°), (2) anti-phase condition (tACS at 5 Hz with a relative phase difference of 180°), and (3) sham condition (tACS at 5 Hz with a relative phase difference of 0° and a stimulation duration of 30 s). For all three conditions, the current was linearly ramped up until the intensity of 1 mA was reached and linearly ramped down to 0 mA at the end of stimulation. Stimulation was applied in a single-blind manner, i.e., subjects were not aware whether they received active tACS or sham stimulation.

**Table 1:** Orthogonalized cross-over design.

	Sequence of stimulation		
Sessions	Group 1 (six participants)	Group 2 (six participants)	Group 3 (six participants)
1	5Hz_0°	5Hz_180°	Sham
2	5Hz_180°	Sham	5Hz_0°
3	Sham	5Hz_0°	5Hz_180°

*All 18 participants received in-phase, anti-phase, and sham stimulation. However, there were three different possible orders. The experimental design is a replication of the design used by Polanía et al. (2012).*

All sessions took place in an acoustically and electromagnetically shielded cabin. To avoid line-frequency interference, all devices inside the cabin were battery-operated. After attachment of the EEG and tACS electrodes, the EEG recordings of the resting condition started with 2 min eyes open and 2 min eyes closed.

#### Experimental Task

**FIGURE 3:**
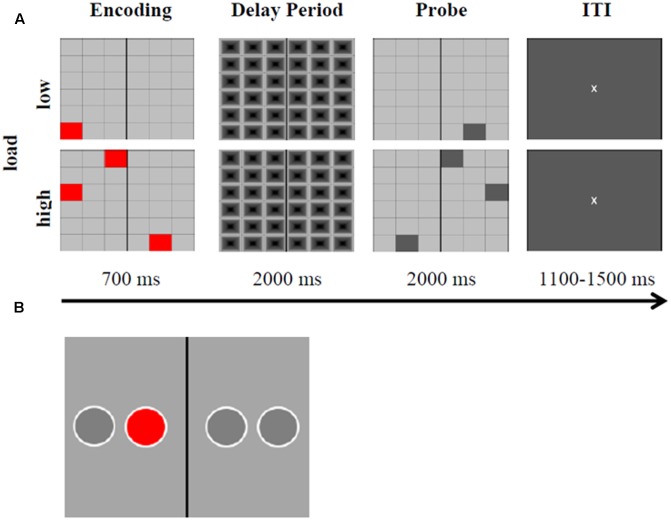
Experimental task and motor response task. **(A)** Representation of the delayed visuospatial match-to-sample task (MtS). Time course with corresponding stimulus material during low and high load, both match and non-match trials are displayed. ITI, inter-trial interval. The MtS was adapted from Griesmayr et al. (2014) and modified respectively. **(B)** Example stimulus of the motor response task. The red circle appeared in each of the four positions randomly. While being stimulated (in-phase, anti-phase, or sham) subjects were instructed to indicate via button press whether the circle appeared to the left or to the right of the vertical black axis. RTs were measured. The task was adapted and modified from Polanía et al. (2012).

A delayed MtS task, adapted from Griesmayr et al. (2014), was used to evaluate two outcome measures of WM performance, i.e., RTs and percentage of accurate responses (**Figure 3A**). A 6 × 6 grid of gray boxes was presented at the center of a screen (19′′ LCD monitor, visual angle of 9.2° × 9.2°, distance to screen: 0.8 m) using E-Prime 2.0 Professional software. Some of the boxes were colored in red, and the subjects’ task consisted in mentally flipping the red boxes on the black vertical axis and keeping this new arrangement in mind after the grid disappeared. Executive demand of WM could either be low, i.e., only one red box, or high, i.e., three red boxes had to be flipped and remembered. After a 2000 ms delay period, a probe stimulus appeared. In 50% of the trials the probe was correct (match) and in 50% of trials it was not correct (non-match). The probe remained for 2000 ms. Participants were asked to indicate with their right index or right middle fingers via button press whether the probe matched the encoding stimulus or not (left arrow key for ‘correct,’ right arrow key for ‘incorrect’). Inter-trial intervals were randomly jittered between 1100 and 1500 ms, with a fixation cross in the middle of the screen. There were 240 trials in total (60 trials pre-stimulation, 120 trials peri-stimulation, and 60 trials post-stimulation). Whereas 50% of all trials were characterized by low executive demand (low load), the other half represented high executive demand trials (high load). The order of presentation was randomized. The black/gray grid during the delay period served the purpose of avoiding color afterimages. Participants were instructed to respond as fast as possible, while maintaining accuracy. Participants completed the MtS task during 7 min before stimulation started, during 14 min while being stimulated, and during 7 min after stimulation had been switched off. A training block was carried out at the beginning of the experiment, until participants achieved performance scores above chance.

#### Motor Response Task

The motor response task was implemented according to Polanía et al. (2012) in order to exclude the possibility of motor cortex stimulation via the Cz return electrode, which could have been responsible for improvements in RT. The task consisted of a red circle appearing in one of four positions which were horizontally spaced on a gray screen and permanently marked. There was a black vertical axis drawn in the middle. Subjects were instructed to press either the left arrow key or the right arrow key (using the same fingers as in the experimental task), depending on whether the red circle appeared to the left or to the right of the vertical axis, respectively. The task consisted of four blocks of 120 trials each. The sequence of circles followed a pseudorandom order, where circles were presented with the same frequency in each position and never in the same position in two subsequent trials. Subjects’ response terminated the current trial. Participants completed the task during 10–12 min (depending on their performance) while being stimulated (see **Figure 3B** for an example stimulus).

#### Electrical Stimulation

**FIGURE 4:**
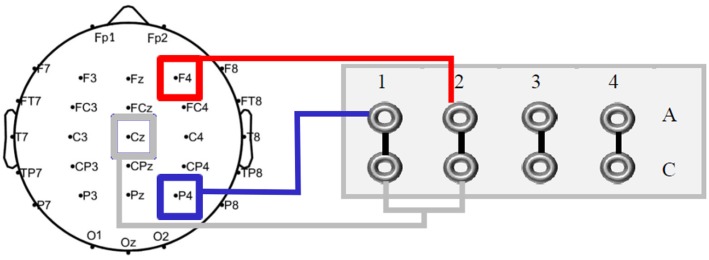
Schematic outline of the multi-channel DC stimulator. Both active electrodes (F4 and P4) were connected to two independent channels. For the return electrode at Cz a cable was electromechanically soldered.

Transcranial alternating current stimulation was applied via two rubber electrodes (5 cm × 5 cm; Neuroconn, Ilmenau, Germany) attached to the head underneath the EEG recording cap, using a battery-operated stimulator system (DC-stimulator plus, Neuroconn, Ilmenau, Germany). The target electrodes were placed over the right prefrontal (F4) and parietal (P4) cortices, with the return electrode at Cz. Given that a multi-channel stimulator system was used, each target electrode could be connected to one independent channel. Thus, in order to maintain stimulation conditions equal, both cables of the corresponding return channels were electromechanically manipulated, resulting in one single merged cable for the return electrode at Cz (see **Figure 4**). In line with results from Griesmayr et al. (2014), the stimulation frequency was 5 Hz (within the theta range). A sinusoidal waveform was applied, without DC offset. Impedance was kept below 10 kΩ. In the first and last 15 s of stimulation, the AC was ramped in and out, respectively. According to standard blinding protocols, current amplitude and frequency were the same in the sham condition as in the experimental conditions, with the difference that AC was only applied for 30 s and afterward turned off automatically in sham. The possibility of phosphene induction within the theta frequency range is rather low and unlikely (Turi et al., 2013). In fact, none of the participants reported phosphenes, neither during the experimental piloting nor during the experimental sessions. Stimulation intensity was set to 1 mA (peak-to-peak), with a total stimulation duration of 26 min for each experimental session. As applied in previous tACS studies (e.g., Neuling et al., 2013), an adaptation of the questionnaire on adverse effects by Brunoni et al. (2011) was used for debriefing.

### Data Analysis

#### Behavioral Data Pre-processing and Analysis

Behavioral data was pre-processed using MATLAB R2014b (The MathWorks, Inc., Natick, MA, United States). For the posterior analysis of RTs, invalid trials were excluded, i.e., RTs > 2000 ms as well as error trials. Subsequently, outliers (> ±2 SD) were removed. As suggested by Baayen and Milin (2015), the proportion of removed data for each data distribution did not exceed 5%. Next, a Shapiro–Wilk parametric hypothesis test of composite normality was run. As expected, none of the distributions was normally distributed. Therefore, RTs were log-transformed. Mean accuracy rates were calculated for each subject during each stimulation condition (in-phase/anti-phase/sham) and for each load condition (low/high).

In order to test if participants were able to tell whether they were actively stimulated or sham stimulated, we conducted a chi-square test. The two categorical variables were STIMULATION (in-phase/anti-phase/sham) and SUBJECTIVE SENSATION (stimulation perceived/no stimulation perceived). Besides, in order to rule out behavioral performance improvements caused by motor cortex stimulation, we calculated a one-way repeated measures (RM) ANOVA on log-transformed RTs during the motor response task [within-subject factor: STIMULATION (in-phase/anti-phase/sham)].

We further conducted a 3x3x2 RM ANOVA on log-transformed RTs as well as accuracy rates assessed during the MtS task. The three within-subject factors were TIME(pre/peri/post), STIMULATION(in-phase/anti-phase/sham), and LOAD(low/high). The factor TIME was included in order to account for a possible learning effect during each experimental session.

#### EEG Data Preprocessing and Analysis

Preprocessing of the electrophysiological data was carried out for resting EEG with eyes closed, using BrainVision Analyzer 2.1. The sequence of preprocessing steps was partly adapted from Miller et al. (2015). First, data were re-referenced to common average. Data were filtered, using a Butterworth zero phase filter (low cut-off: 0.5 Hz, high cut-off: 70 Hz, Slope: 24 db/Oct, Notch: 50 Hz). Next, an ocular correction ICA (independent component analysis) was performed to correct artifacts caused by eye movements and muscle activity. Data were then manually inspected for remaining eye and muscle artifacts. A fast Fourier transformation (FFT) with a 10% Hanning window (frequency resolution 0.488 Hz) was applied to the data. For all further statistical analysis MATLAB R2014b (The MathWorks, Inc., Natick, MA, United States) and SPSS 20.0 (IBM, Corp., Armonk, NY, United States) were used.

An exploratory EEG analysis of power peaks within the delta (0.5–4 Hz), theta (4–8 Hz), alpha (8–12 Hz), and beta (12–30 Hz) frequency bands was carried out. For all electrodes, peaks of spectral power were calculated algorithmically by determining the maximum amplitude within each frequency band.

A 2x3x18 RM ANOVA was run for each frequency band. The three within-subject factors were PRE_POST, STIMULATION (in-phase/anti-phase/sham), and ELECTRODE.

A region of interest (ROI) was defined consisting of the following electrodes: Fz, F8, Pz, P8, and Oz. These EEG electrodes were the ones located within the immediate vicinity of our stimulation electrodes. We expected an EEG power increase in both the in-phase and the anti-phase condition, as the tACS mechanism remains identical in both stimulation conditions. Both conditions (in-phase and anti-phase) only differ with respect to timing. During in-phase tACS, F4, and P4 receive stimulation simultaneously. During anti-phase stimulation, F4 and P4 receive stimulation with a time lag (180° relative phase angle). The measured EEG power post stimulation only reflects local power changes, i.e., power increase/decrease of underlying neural populations. Hence, an EEG power analysis does not take into account the timing aspect of stimulation, but changes in EEG power can be expected in active tACS (in-phase and anti-phase) compared to sham (Zaehle et al., 2010; Neuling et al., 2013). Therefore, we computed two reduced 2x2x5 RM ANOVAs (PRE_POST, STIMULATION, ROI). The within-subject factor STIMULATION in these analyses comprised the levels in-phase/sham and anti-phase/sham, respectively (Supplementary Figure 1 shows the non-linear effect of stimulation which justifies the use of a 2x2x5 ANOVA).

Mauchly’s test of sphericity was applied to every analysis and Greenhouse–Geisser or Huynh-Feldt corrections were performed when sphericity was violated. Subsequent *post hoc* tests were Bonferroni corrected.

In a further step, individual Δθ (the difference between the stimulation frequency of 5 Hz and each participant’s peak frequency within the theta range) was analyzed in order to determine the individual theta peak shift toward or away from the stimulation frequency after being stimulated. For this purpose, we first computed theta peak power algorithmically on an individual level before as well as after tACS. Next, we determined the specific theta frequency of this peak power value. The output was one theta peak frequency value for each participant, which was calculated by averaging over five ROI electrodes (Fz, F8, Pz, P8, Oz). Next, Δθ was computed by subtracting 5 Hz from the aforementioned theta peak frequency value. Hence, for each participant six Δθ values were obtained, i.e., one pre tACS and one post tACS, for in-phase, anti-phase, and sham stimulation. Finally, we assessed the strength of the linear association between Δθ and log-transformed RTs by means of a Pearson correlation (Bonferroni corrected).

## Results

### Behavioral Data

When asked whether stimulation was real or sham, participants were not able to tell reliably [χ^2^(2) = 5.85, *p* > 0.05]. Moreover, no differences in RTs between stimulation conditions (in-phase/anti-phase/sham) were found in the motor response task, as evidenced by a one-way RM ANOVA [*F*(2,32) = 0.40, *p* > 0.05]. Hence, a behaviorally facilitating stimulation of the motor cortex can be ruled out.

**FIGURE 5:**
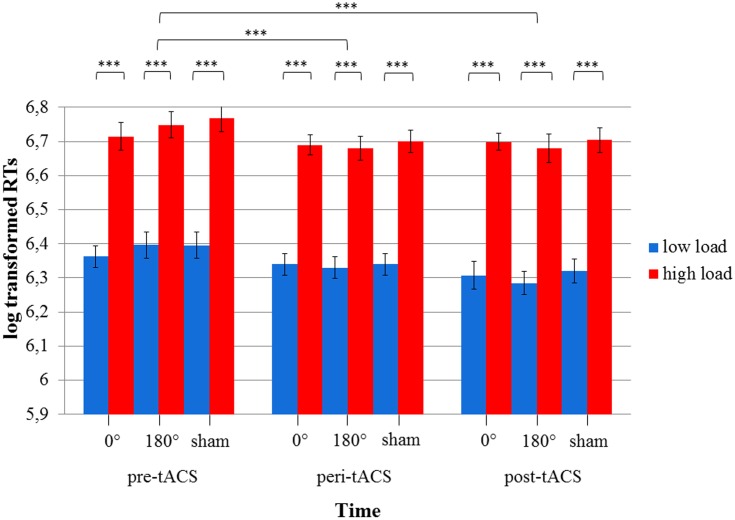
Diagram of log-transformed reaction times (RTs) during the visuospatial match-to-sample task. Significant differences between low and high load in each of the three stimulation conditions (0° = in-phase, 180° = anti-phase, and sham) for pre-, peri-, and post-tACS as well as significant differences between pre-, peri-, and post-tACS are shown. (^∗∗∗^*p* < 0.001) Standard error bars are displayed.

**FIGURE 6:**
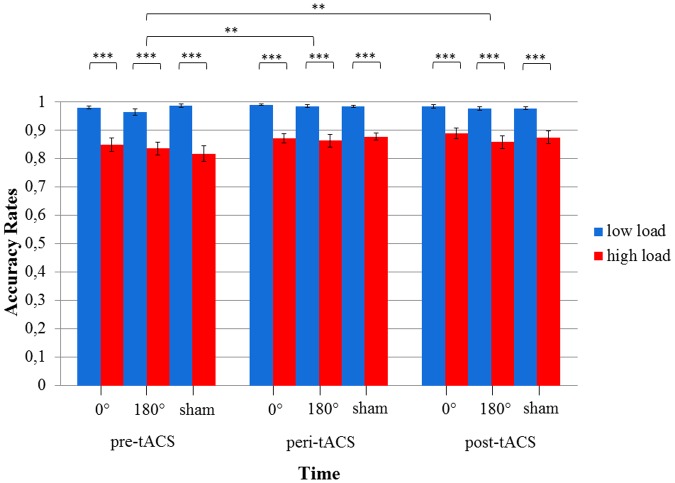
Diagram of accuracy rates during the visuospatial match-to-sample task. Significant differences between low and high load in each of the three stimulation conditions (0° = in-phase, 180° = anti-phase, and sham) for pre-, peri-, and post-tACS as well as significant differences between pre-, peri-, and post-tACS are shown. (^∗∗∗^*p* < 0.001; ^∗∗^*p* < 0.05) Standard error bars are displayed.

The 3x3x2 RM ANOVA of log-transformed RTs (assessed during the MtS task) with the within-subject factors TIME, STIMULATION and LOAD showed a main effect for TIME [*F*(2,34) = 24.1, *p* < 0.0001] as well as LOAD [*F*(1,17) = 623.1, *p* < 0.0001], i.e., RTs during the high load condition were larger than during the low load condition. There were no statistically significant effects for STIMULATION or any of the factor interactions (all *p*s > 0.05) (see **Figure 5**). RTs improved over the course of the experimental session (pre-peri-post), i.e., the fastest responses were measured post-stimulation. Importantly, this improvement could be observed across all three stimulation conditions. With respect to accuracy rates, the 3x3x2 RM ANOVA yielded similar results: a main effect for TIME [*F*(2,34) = 6.83, *p* < 0.05] as well as LOAD [*F*(1,17) = 87.6, *p* < 0.0001], and no statistically significant effects for STIMULATION or any of the factor interactions (all *p*s > 0.05) (see **Figure 6**).

### EEG Data

**FIGURE 7:**
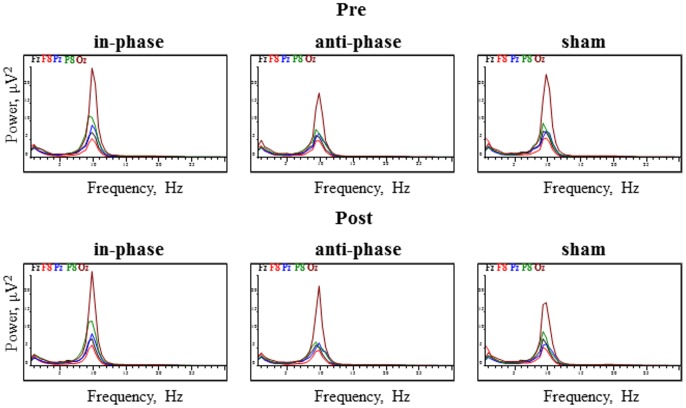
Power spectrograms of resting EEG before and after stimulation for the three stimulation conditions (in-phase, anti-phase, and sham). EEG power (in μV^2^) for the five electrodes within direct vicinity of the stimulation sites, i.e., Fz, F8, Pz, P8, and Oz is displayed.

The 2x3x18 RM ANOVA with the within-subject factors PRE_POST, STIMULATION, and ELECTRODE revealed a main effect for ELECTRODE in every analyzed frequency band [delta: *F*(3,53) = 8.49, *p* < 0.0001, theta: *F*(3,53) = 8.4, *p* < 0.0001, alpha: *F*(2,26) = 9.33, *p* < 0.05, beta: *F*(3,45) = 17.9, *p* < 0.0001]. There were no other significant main effects (all *p*s > 0.05). Regarding the PRE_POST × STIMULATION interaction, no frequency band showed significant effects [delta: *F*(2,34) = 0.18, *p* > 0.05; theta: *F*(1,23) = 0.54, *p* > 0.05; alpha: *F*(1,23) = 1.6, *p* > 0.05; beta: *F*(1,21) = 0.6, *p* > 0.05]. Moreover, the analysis did not yield any significant PRE_POST × STIMULATION × ELECTRODE interactions for the delta [*F*(34,578) = 0.58, *p* > 0.05], theta [*F*(34,578) = 1.05, *p* > 0.05], and beta [*F*(34,578) = 1.2, *p* > 0.05] frequency bands. However, in the alpha frequency band, we observed a significant interaction: [*F*(34,578) = 1.8, *p* < 0.05]. **Figure 7** displays power spectrograms of resting EEG for the three stimulation conditions (in-phase, anti-phase, and sham) before and after stimulation.

To further test the effect of stimulation (i.e., stimulation conditions vs. sham) on the alpha peak power, we run two separate ANOVAs (in-phase vs. sham, and anti-phase vs. sham) over five ROI electrodes (Fz, F8, Pz, P8, Oz). When comparing in-phase and sham stimulation conditions, a significant interaction PRE_POST × STIMULATION was found: *F*(1,17) = 5.7, *p* < 0.05. Interestingly, *post hoc* paired samples *t*-tests (with the dependent variable being the mean of the aforementioned ROI electrodes) revealed that the pre vs. post contrast was only significant for sham stimulation [*t*(17) = 2.2, *p* < 0.05] but not for in-phase stimulation [*t*(17) = -0.09, *p* > 0.05]. The alpha peak power significantly decreased from pre to post after sham stimulation, whereas it stayed constant for in-phase stimulation. The 2x2x5 RM ANOVA (pre/post, anti-phase/sham, Fz/F8/Pz/P8/Oz) did not yield any statistically significant interactions (all *p*s > 0.05).

**FIGURE 8:**
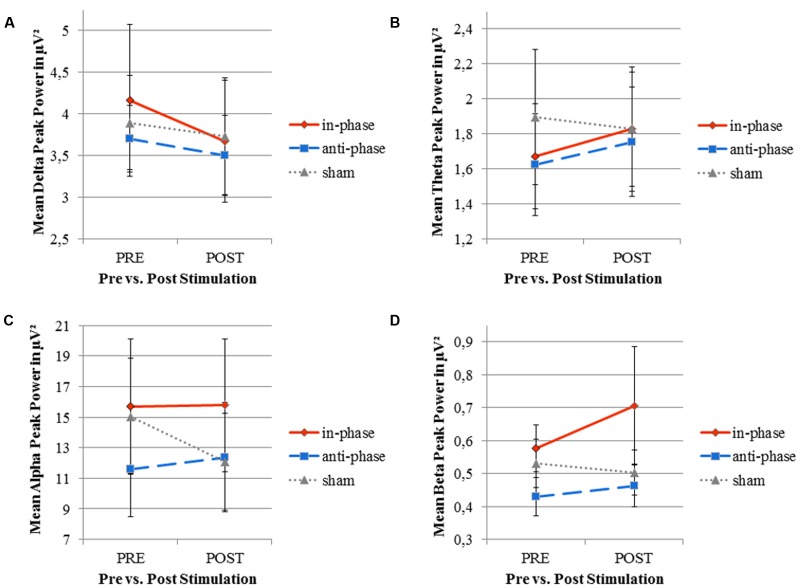
Mean peak power of resting EEG (delta, theta, alpha, and beta frequency bands) before and after stimulation for the three stimulation conditions (in-phase, anti-phase, and sham). **(A)** Delta frequency range (0.5-4 Hz). **(B)** Theta frequency range (4-8 Hz). **(C)** Alpha frequency range (8-12 Hz). **(D)** Beta frequency range (12-30 Hz). Peak power (in μV^2^) was averaged across five electrodes within direct vicinity of the stimulation sites, i.e., Fz, F8, Pz, P8, and Oz. Standard error bars are displayed.

Contrary to our experimental hypothesis, there were no significant changes in the EEG power spectrum from pre to post for the stimulation frequency (theta band) (see **Table 2** for details). **Figure 8** illustrates mean peak power values pre as well as post-stimulation for the delta, theta, alpha, and beta range.

**Table 2:** Mean theta peak power values, standard errors, and confidence intervals for pre/post and stimulation conditions (in-phase/anti-phase/sham).

				95% Confidence interval	
Condition	Stimulation	Mean	*SE*	Lower bound	Upper bound
Pre	In-phase	1.67	0.30	1.04	2.31
	Anti-phase	1.63	0.29	1.02	2.24
	Sham	1.90	0.39	1.08	2.71
Post	In-phase	1.83	0.33	1.14	2.52
	Anti-phase	1.76	0.31	1.10	2.42
	Sham	1.83	0.36	1.08	2.58

*EEG electrodes in immediate vicinity of stimulation electrodes, i.e., Fz, F8, Pz, P8, Oz were used in order to compute mean peak power values.*

### Correlation Analysis of EEG and Behavioral Data

**FIGURE 9:**
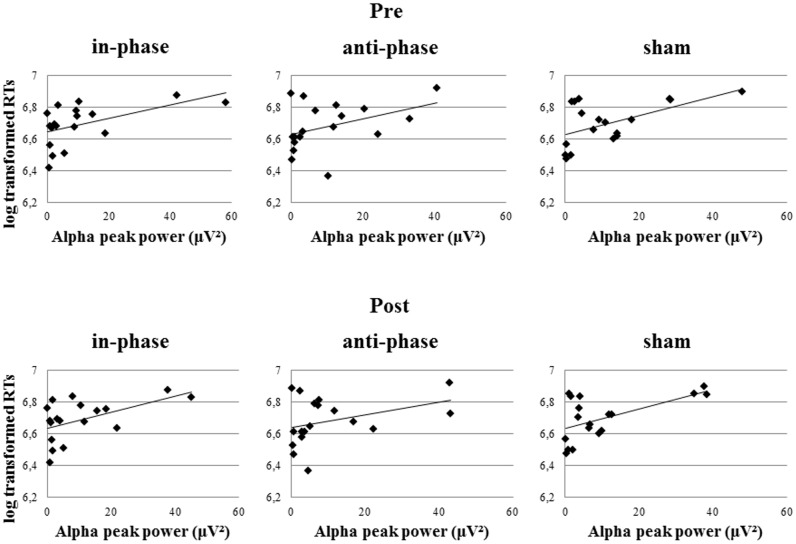
Pearson correlations of resting EEG alpha peak power (before and after stimulation) and log transformed RTs during the MtS task during the three stimulation conditions (in-phase, anti-phase, and sham).

Markedly, alpha peak power positively correlated with RTs, before as well as after the stimulation (see **Table 3**). This effect was observed in the in-phase and sham conditions, whereas the trend did not reach significance in the anti-phase condition. In other words, the stronger the alpha power, the slower participants responded during the behavioral task. Alpha power values before the task during the resting period could predict performance in the MtS task, as revealed by a bivariate Pearson correlation between log-transformed RTs and alpha peak power before the stimulation (in-phase: *r* = 0.52, *p* < 0.05; anti-phase: *r* = 0.4, *p* > 0.05; sham: *r* = 0.55, *p* < 0.05) (see **Figure 9**).

**Table 3:** Correlations between alpha peak power and log transformed RTs for pre/post and stimulation conditions (in-phase/anti-phase/sham).

	Stimulation		
Condition	In-phase	Anti-phase	Sham
Pre	*r* = 0.52, *p* = 0.03	*r* = 0.40, *p* = 0.11	*r* = 0.55, *p* = 0.02
Post	*r* = 0.52, *p* = 0.03	*r* = 0.36, *p* = 0.15	*r* = 0.57, *p* = 0.01

**FIGURE 10:**
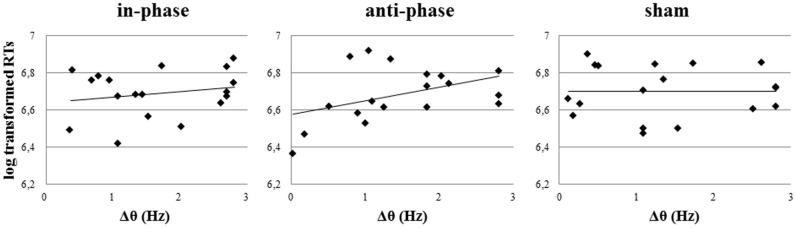
Pearson correlations of Δθ post-stimulation and log transformed RTs during the MtS task during the three stimulation conditions (in-phase, anti-phase, and sham).

Furthermore, there was a significant positive correlation between Δθ and log transformed RTs, which could only be observed post-stimulation and only in the anti-phase condition (in-phase: *r* = 0.27, *p* > 0.05; anti-phase: *r* = 0.6, *p* < 0.05; sham: *r* = -0.13, *p* > 0.05) (see **Figure 10**). The closer participants’ individual theta peak frequency was to the stimulation frequency of 5 Hz, the faster they responded during the behavioral task. On the other hand, Δθ values before stimulation did not significantly correlate with log transformed RTs during the MtS task (in-phase: *r* = 0.14, *p* > 0.05; anti-phase: *r* = 0.24, *p* > 0.05; sham: *r* = -0.2, *p* > 0.05).

## Discussion

### Lack of Frequency-Specific EEG Aftereffects

The present exploratory study did not show any EEG power enhancement for the tACS-targeted theta frequency band. This finding is in line with results recently published by Wischnewski et al. (2016), who did not observe any changes in resting EEG after theta tACS compared to sham. However, these authors reported a significant decrease in theta-beta EEG ratios at frontal recording sites following active tACS.

Our analyses of other frequency bands revealed, nonetheless, a frequency-unspecific effect in the alpha frequency band. We report a significant drop of EEG alpha power in sham, whereas alpha power remained equal from pre to post in the in-phase and anti-phase conditions. EEG power did not differ significantly between anti-phase and in-phase tACS in any frequency band.

With regard to memory processes, Klimesch et al. (2006) stated that resting or reference alpha power was positively related to participants’ performance. The results of the present study – log transformed RTs during the task and alpha power before stimulation correlate positively – confirm this finding by Klimesch and colleagues. This suggests that resting alpha power before stimulation is a good predictor of WM performance.

Interestingly, Klimesch and colleagues also observed that event-related alpha desynchronization (ERD), reflected by small power during the actual task, was associated with good performance (Klimesch et al., 2006). Moreover, in an earlier study, Klimesch (1999) has found evidence that the extent of alpha ERD is related to task demands, i.e., as the task becomes more difficult, alpha power drops and theta power increases. Furthermore, the transition between theta synchronization and alpha desynchronization is subject to large inter-individual variability (Klimesch, 1999). Although individual differences with regard to alpha peak frequency strongly depend on age, even for age-matched subjects a considerable inter-individual variability in alpha frequency has been observed (Doppelmayr et al., 1998). Klimesch et al. (1990, 1993) showed that these inter-individual differences in alpha frequency are mainly due to inter-individual differences in memory performance.

Nevertheless, the dissociation between tonic (resting/reference) and phasic (event-related) alpha power provides a tangible explanation for the results of the present experiment. Theta tACS may have increased alpha power on a phasic level in the two active stimulation conditions during the actual WM task, which could have deteriorated participants’ behavioral performance, masking the effects of theta power enhancement. The significant decrease in alpha power in the sham condition could be taken as evidence for the alpha desynchronization, crucial for good WM performance.

### Behavioral Findings

The absence of electrophysiological effects in the theta range was paralleled by an absence of behavioral effects between conditions. Contrary to our initial experimental hypotheses, the present study did not show any significant differences in RTs or accuracy rates between the two stimulation conditions (in-phase and anti-phase). Based on these findings, we cannot draw concrete conclusions about the role of theta phase synchronization or desynchronization in WM processes and if tACS can be used to differentiate between in-phase and anti-phase phase-locking between brain areas.

With our analyses we also addressed a possible learning effect. Participants’ behavioral performance (RTs and accuracy rates) improved significantly over the course of each experimental session (pre-peri-post). Yet, this behavioral improvement was similar in all stimulation conditions and we can thus rule out a tACS facilitated learning boost.

### Correlation Analysis of EEG and Behavioral Data

Notably, the present study showed that the smaller Δθ in a given subject after anti-phase stimulation, the more behavioral performance during the WM task was facilitated (i.e., faster RTs). This finding is in line with results from Griesmayr et al. (2014) and confirms the correct choice of a 5 Hz target frequency for the specific visuospatial WM paradigm used in the present study. Interestingly, Δθ before stimulation could not predict RTs during the task, but Δθ after stimulation could. The closer a participant’s individual theta peak frequency had shifted toward the stimulation frequency after stimulation, the faster her RT during the task. We suggest two possible interpretations for this finding: (A) Frequency shifts were due to entrainment by tACS. (B) Stimulation at individual peak power frequency was not relevant in our experiment, as otherwise Δθ before stimulation should have been a good predictor of RTs. In line with these findings, Helfrich et al. (2014) pointed out that neither baseline power nor the iAPF reliably predicted whether 10 Hz tACS resulted in successful entrainment. Behavioral data by Cecere et al. (2015) further support this interpretation.

### Failed Replication of tACS Phase Manipulation

The present study attempted to replicate the ‘synchronization-desynchronization’ tACS setup, originally introduced by Polanía et al. (2012). However, three main caveats to this earlier study may underlie the inconsistency of findings between the study by Polanía and colleagues and the present study. First, Polanía and colleagues did not measure participants’ EEG, neither before, during nor after the tACS experiment and could thus not provide any direct evidence for enhancement of synchronous brain oscillations in the theta band. Second, electromechanical limitations of the stimulation device used by Polanía and colleagues caused a fundamental methodological problem. Apart from the relative phase angle of stimulation (0 or 180°), the authors could not rule out the possibility that their ‘synchronized’ group differed in one more important parameter from their ‘desynchronized’ group, namely amplitude. Due to the specific electrode setup, it is possible that the ‘synchronized’ group was stimulated with a different intensity than the ‘desynchronized’ group, which might have caused differences between groups. Recently, Strüber et al. (2013) used a similar protocol successfully with 40 Hz tACS. Nonetheless, they applied two return electrodes in their ‘in-phase’ condition – one on each hemisphere – and only one return electrode in their ‘anti-phase’ condition. Since sinusoidal currents constantly switch between active and return electrodes, again, it can be questioned whether the two stimulation conditions used by Strüber and colleagues were comparable after all. Third, a recent review by Thut et al. (2017) raised the issue of the direction of current flow in Polanía and collegues’ electrode setup. Whereas the direction of current flow in the ‘synchronized’ group was F3-Cz/Cz-F3 and P3-Cz/Cz-P3, the direction in the ‘desynchronized’ group was F3-P3/P3-F3. The present study was specifically designed to overcome these technological limitations. We used a multichannel stimulator with in-house electromechanical adjustments of the stimulation electrodes which enabled us to control for stimulation intensities (i.e., 1 mA peak-to-peak) as well as for the direction of current flow (i.e., F4-Cz/Cz-F4 and P4-Cz/Cz-P4).

### Limitations and Future Directions

With regard to our EEG analyses, a first limitation of the present study lies in the fact that we did not directly take into account inter-individual differences due to following the convention of analyzing fixed frequency bands. Since, on an individual level, theta frequency varies as a function of alpha frequency, this limitation could be overcome in the future by using alpha frequency as a reference point for calculating other frequency bands as suggested by Doppelmayr et al. (1998).

A second limitation is the lack of online-EEG recordings during tACS. Unfortunately, such simultaneous tACS-EEG recordings are subject to strong artifacts, which impose a substantial drawback to neuroscientific research in the field of non-invasive brain stimulation. Very recently an increasing number of studies have tried to overcome this constraint by implementing complex mathematical algorithms, including principal component analysis (Fehér and Morishima, 2016) or superposition of moving averages (Kohli and Casson, 2015) as well as alternative stimulation paradigms, e.g., sawtooth waves (Dowsett and Herrmann, 2016). Despite these efforts, it has been pointed out by Noury et al. (2016) that physiological processes, such as heartbeat and respiration, modulate stimulation artifacts in a non-linear manner. Hence, until now current techniques have failed to remove artifacts entirely. Nonetheless, the concurrent use of tACS and neuroimaging methods such as MEG (Neuling et al., 2015), EEG (Helfrich et al., 2014) or fMRI (Vosskuhl et al., 2016) and the possibility of source reconstruction and mapping tACS entrained cortical oscillations (Witkowski et al., 2016) might yield crucial insights into the online effects of electrical brain stimulation in the future.

A third limitation of our study lies in the choice of stimulation frequency, i.e., 5 Hz. Even though Polanía et al. (2012) and Griesmayr et al. (2014), for instance, have shown that fronto-parietal theta coupling might constitute a key mechanism in WM processes, phase relationships in other frequency bands have as well been found to play crucial roles. Alpha-band oscillations might not only be linked to inhibition (i.e., attention suppression), but also to the selection of stored information (Klimesch, 2012). Furthermore, Bonnefond and Jensen (2012) reported stronger alpha power increase and phase adjustment within occipito-temporal brain areas prior to anticipated distractors as a possible protective mechanism of WM maintenance. Roux and Uhlhaas (2014) further proposed the idea that rhythmic activity at different frequency bands may reflect functional task-dependent differences in WM processes. On the one hand, the authors advocate the involvement of theta oscillations in the sequential coding of WM items. On the other hand, they highlight the occurrence of alpha activity during visual and/or spatial tasks that depend upon the maintenance of simultaneously presented items. Nonetheless, such a visuospatial task with simultaneous presentation of WM material also revealed fronto-parietal phase synchronization within the beta and gamma frequency bands (Babiloni et al., 2004). Besides, Klimesch et al. (2004) have pointed out that alpha-theta phase locking is associated with semantic and WM performance. Had we thus chosen a different stimulation frequency for the present study, we would have possibly observed different behavioral and electrophysiological effects. In the future, more sophisticated protocols could offer the prospect of multi-frequency stimulation in order to tackle research questions regarding cross-frequency coupling, as suggested by Novembre et al. (2017).

## Conclusion

The synchronization of oscillatory phases between distant cortical areas seems to be a fundamental neural mechanism, which has proven to be highly relevant for process binding, large-scale communication and integration of neural networks (Womelsdorf et al., 2007; Siegel et al., 2012). The present exploratory study intended to investigate whether such synchronous oscillations are a mere epiphenomenon or actually serve a causal purpose in WM. TACS is widely considered a valuable method in cognitive neuroscience (Herrmann et al., 2013). However, how tACS precisely entrains neural oscillations is still subject to an ongoing debate (Thut et al., 2011; Underwood, 2016). The results of this study emphasize that the modulation of intrinsic neural oscillations by tACS is not simple and one-dimensional. While tACS has repeatedly been shown to impact neural oscillations in a frequency-specific manner (e.g., Zaehle et al., 2010) with subsequent effects on sensation and behavior (Feurra et al., 2011), the absence of frequency-specific effects on neural oscillations (Brignani et al., 2013; for review see Veniero et al., 2015), as well as the absence of behavioral effects on WM performance have been reported elsewhere (Santarnecchi et al., 2016). With the well-known negative publication bias in mind (Fanelli, 2011; Bikson et al., 2014), the negative results of this study should be seen as a cautionary reminder that the precise mechanisms of how tACS impacts neuronal circuits are still unclear. Recently, much work has targeted these precise mechanisms and effects (Neuling et al., 2012; Datta et al., 2013; for review see Ling et al., 2016; Thut et al., 2017) with the aim to ensure tighter control of experimental set-ups and stimulation parameters (Datta et al., 2009; Bikson et al., 2010; Dmochowski et al., 2011). Once our understanding of tACS accounts, for instance, for both frequency-specific and frequency-unspecific effects, tACS may reach its full potential as an experimental tool to causally test hypotheses on principles of neural oscillations.

## Author Contributions

M-LK, VM, and CS designed the study, M-LK acquired and analysed the data. M-LK, VM, and CS discussed the results, and M-LK wrote the article. All authors read and approved the final version of the manuscript.

## Conflict of Interest Statement

The authors declare that the research was conducted in the absence of any commercial or financial relationships that could be construed as a potential conflict of interest.
