# Supplementary material for: Frequency-Unspecific Effects of θ-tACS Related to a Visuospatial Working Memory Task
Source: Front Hum Neurosci. 2017 Jul 12;11:367. doi: 10.3389/fnhum.2017.00367 (PMC5506205; doi:10.3389/fnhum.2017.00367)
Supplement: Supplementary file 1 [file Image_1.pdf]

frontiers

Supplementary Material

Frequency-Unspecific Effects of θ-tACS Related to a Visuospatial Working Memory Task

Maria-Lisa Kleinert*, Caroline Szymanski, and Viktor Müller*

*** Correspondence:**

Corresponding Author: Maria-Lisa Kleinert: kleinert@mpib-berlin.mpg.de

Viktor Müller: vmueller@mpib-berlin.mpg.de

1 Supplementary Figures

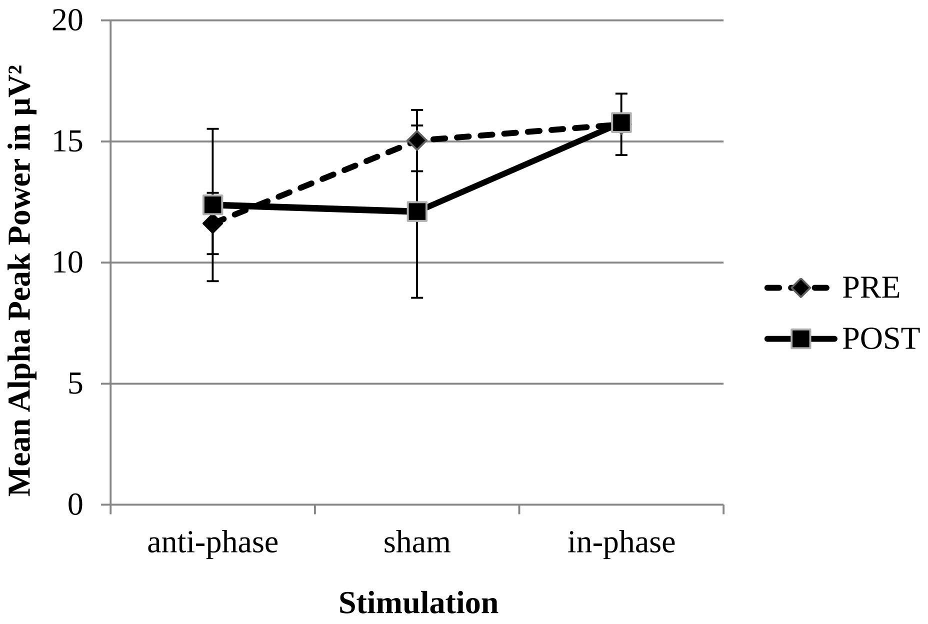

Supplementary Figure 1. Mean alpha peak power of resting EEG in the three stimulation conditions (anti-phase, sham and in-phase) before and after stimulation. Peak power (in µV²) was averaged across five electrodes within direct vicinity of the stimulation sites, i.e., Fz, F8, Pz, P8, and Oz. Standard error bars are displayed.
